# Zinc Toxicity: Understanding the Limits

**DOI:** 10.3390/molecules29133130

**Published:** 2024-07-01

**Authors:** Hannah Schoofs, Joyce Schmit, Lothar Rink

**Affiliations:** Institute of Immunology, Medical Faculty, RWTH Aachen University, Pauwelstrasse 30, 52074 Aachen, Germany; hannah.schoofs@rwth-aachen.de (H.S.); joyce.schmit@rwth-aachen.de (J.S.)

**Keywords:** toxicity, zinc, exposure, copper, upper intake level, recommendation

## Abstract

Zinc, a vital trace element, holds significant importance in numerous physiological processes within the body. It participates in over 300 enzymatic reactions, metabolic functions, regulation of gene expression, apoptosis and immune modulation, thereby demonstrating its essential role in maintaining overall health and well-being. While zinc deficiency is associated with significant health risks, an excess of this trace element can also lead to harmful effects. According to the World Health Organization (WHO), 6.7 to 15 mg per day are referred to be the dietary reference value. An excess of the recommended daily intake may result in symptoms such as anemia, neutropenia and zinc-induced copper deficiency. The European Food Safety Authority (EFSA) defines the tolerable upper intake level (UL) as 25 mg per day, whereas the Food and Drug Administration (FDA) allows 40 mg per day. This review will summarize the current knowledge regarding the calculation of UL and other health risks associated with zinc. For example, zinc intake is not limited to oral consumption; other routes, such as inhalation or topical application, may also pose risks of zinc intoxication.

## 1. Introduction

Zinc is an essential trace element, as it plays a crucial part in various physiological processes within the human body. Zinc is involved in a multitude of cellular processes, including enzyme function, regulation of gene expression, DNA metabolism, cell differentiation and proliferation, signal transduction, the immune response and the regulation of cell death [1,2,3].

Given the numerous functions of zinc in signal transduction, the regulation of intra- and extracellular zinc concentration is tightly modulated. A variety of proteins are involved in the maintenance of zinc homeostasis. The two major protein families involved in cellular zinc homeostasis are the SLC39 family, known as Zrt- and Irt-like proteins (ZIP), and the SLC30 family, referred to as Zn transporters (ZnT). The SLC39/ZIP transporters encode proteins ZIP1 to ZIP14. The SLC30/ZnT family comprises 10 proteins, namely, ZnT1 to ZnT10 [4,5]. The ZIP transporters facilitate the influx of ions into the cytoplasm. ZIP transporters possess eight transmembrane domains and contain a histidine-rich intracellular loop that is involved in zinc binding and its transport. ZnT proteins facilitate the efflux of zinc ions from the cytoplasm into other cellular compartments or into the extracellular space. ZnT proteins contain six transmembrane domains with an intramembrane zinc binding site that is involved in zinc binding. Both ZIP and ZnT proteins are subject to regulation in response to fluctuations in zinc levels. A disruption of zinc homeostasis can contribute to the development of zinc deficiency or toxicity or toxicity by other metals [6,7]. Thus, ensuring adequate zinc bioavailability and maintaining zinc homeostasis are vital for overall health and well-being. Zinc bioavailability is defined as the proportion of zinc that must be continuously absorbed from the diet, given that there is no dedicated compartment for zinc storage [8]. In addition to the dietary intake of zinc, the efficiency of absorption also plays a role. The absorption of zinc can be influenced by the presence of other trace elements, such as copper and iron [9].

Therefore, this review aims to provide a comprehensive summary of the current knowledge on zinc bioavailability and zinc toxicity, with a particular focus on the impacts of acute zinc poisoning, as well as the consequences of prolonged (chronic) exposure to high levels of zinc. Furthermore, an overview of the role of zinc in various diseases and health conditions is provided, with an emphasis on the reciprocal relationship between zinc and copper [10].

## 2. Recommendation for Zinc Intake

Several institutions have developed dietary zinc intake guidelines. The U.S. Department of Agriculture’s (USDA) Food Data Central provides a comprehensive listing of the zinc content of various foods [11]. The zinc content, phytate levels and phytate-to-zinc ratios of common foods are displayed in Table 1. Phytate, which is present in plant-based foods, has a negative effect on the bioavailability of zinc in food, as it can bind trace elements, such as zinc. Consequently, the recommended daily intake of zinc increases in proportion to the phytate content of the consumed food [12]. Seafood and meat products are rich in zinc, suggesting that a diet including these foods may help maintain adequate zinc levels, especially given their very low phytate levels in comparison with various legumes, seeds or grains. Such foods are typically classified as having a moderate or high phytate level. It can therefore be concluded that the required intake of zinc increases. This also leads to the assumption that vegetarians and vegans are more likely to have a zinc deficiency, as their zinc sources contain higher phytate levels and, therefore, the actual required zinc intake increases. According to the Academy of Science in the United States, the recommended dietary allowance (RDA) for zinc intake states that 11 mg/day for men and 8 mg/day for women are sufficient [13]. Nevertheless, it is important to note that different countries have different recommendations, as illustrated in Table 2. The EFSA specifies the population reference intake (PRI) for men ranges from 9.4 to 16.3 mg/day and from 7.5 to 12.7 mg/day for women [14]. In addition, the recommendations of Germany and France were considered separately. For Germany, the German Nutrition Society (DGE) recommends 11 to 16 mg/day zinc for men and 7 to 10 mg/day for women, depending on the intake of phytate [15]. The French Agency for Food, Environmental and Occupational Health and Safety (ANSES) sets the values for PRI for men at 9.4 to 14 mg/day and for women at 7.5 to 11 mg/day, depending on the phytate intake level [16]. Additionally, the recommended nutrient intake (RNI) for the United Kingdom is listed in Table 2. The RNIs are set at 9.5 mg/day for men and 7 mg/day for women [17]. The Japanese Ministry of Health, Labour and Welfare recommends 10 mg/day for men and 8 mg/day for women, which is comparable to the values observed in the United States [18].

As the Indian and Chinese populations make up a large part of the world’s population, the recommendations for zinc intake were also considered. According to the Indian Council of Medical Research (ICMR), the RDA of zinc includes 17 mg/day for men and 13.2 mg/day for women [19]. In China, the RNI for zinc is set at 12.5 mg/day for men and 7.5 mg/day for women [20].

In Table 3, recommendations from the WHO are shown, which include subdivisions for different bioavailabilities of zinc and for different age groups and genders [21]. In Table 4, the population reference intake of zinc for infants and children according to EFSA is shown. Table 5 presents the population reference intake of zinc for adults, stratified by phytate levels as determined by the EFSA.

**Table 1:** Amount of zinc present in food [11,22,23,24,25]. Phytate-to-zinc molar ratio was calculated based on the following equation (mg phytate/660 kDa)/(mg zinc/65 kDa).

Food	Amount of Zinc (mg/100 g)	Phytate Level (mg/100 g)	Phytate-to-Zinc Ratio
Seafood and meat			
Oyster (cooked)	16–91; dependent on species and preparation method	0	0
Beef (cooked, lean)	4.6	0	0
Chicken (cooked, breast)	1.5	0	0
Legumes			
Lentils (raw)	3.3	588.7	17.8
Chickpeas (raw)	2.8	458.2	16.1
Red kidney beans (raw)	2.8	888	31.3
Soybeans	4.89	222	4.5
Nuts and Seeds			
Peanuts (raw)	3.27	771.5	23.2
Hemp seeds	9.9	280	2.8
Cashew nuts	5.6	498	8.76
Whole grains			
Wheat germ	3.2	391	12.0
Oats	0.8	116	14.6
Quinoa	1.1	118	10.6
Dairy products			
Cheese, Cheddar	3.1	0	0
Milk, whole	0.4	0	0

**Table 2:** Recommendations for zinc intake for men and women worldwide.

Country	RDA/PRI/RNI (mg/Day)	
	Men	Women
China	12.5	7.5
India	17	13.2
Europe	9.4 to 16.3	7.5 to 12.7
United States	11	8
Japan	10	8
Germany	11 to 16	7 to 10
France	9.4 to 14	7.5 to 11
United Kingdom	9.5	7

**Table 3:** Recommended nutrient intake (RNI) for zinc (mg/day) according to WHO depending on zinc bioavailability [21].

Group	Low Bioavailability * (15%)	Moderate Bioavailability * (30%)	High Bioavailability * (50%)
0–6 months	6.6 ^b^	2.8 ^b^	1.1 ^a^
7–12 months	8.4	4.1	0.8 ^a^/2.5
1–3 years	8.3	4.1	2.4
4–6 years	9.6	4.8	2.9
7–9 years	11.2	5.6	3.3
10–18 years, male	17.1	8.6	5.1
10–18 years, female	14.4	7.2	4.3
Men, 19+	14.0	7.0	4.2
Women, 19+	9.8	4.9	3.0

^a^ RNI for dietary zinc exclusively for human-milk-fed infants. ^b^ RNI for dietary zinc for formula-fed infants. * Low availability equals a phytate–zinc molar ratio of >15, moderate availability equals to a ration between 5 and 15 and high availability to a phytate–zinc ratio <5.

**Table 4:** Population reference intake (PRI) for zinc for infants and children [14].

Group	PRI (mg/Day)
7–11 months	2.9
1–3 years	4.3
4–6 years	5.5
7–10 years	7.4
11–14 years	10.7
15–17 years, male	14.2
15–17 years, female	11.9

**Table 5:** Population reference intake (PRI) for zinc according EFSA depending on phytate intake for adults [14].

Group	Level of Phytate Intake (mg/Day)	PRI for Zinc (mg/Day)
18 years+, male	300	9.4
	600	11.7
	900	14.0
	1200	16.3
18 years+, female ^a^	300	7.5
	600	9.3
	900	11.0
	1200	12.7

^a^ +1.6 mg/day for pregnant women and +2.9 mg/day for lactating women.

## 3. Groups at Risk for Zinc Inadequacy

Certain groups are more susceptible to zinc shortage or toxicity because of a variety of factors, including lifestyle choices, dietary practices and underlying medical conditions.

### 3.1. Groups at Risk for Zinc Intoxication

**Figure 1:**
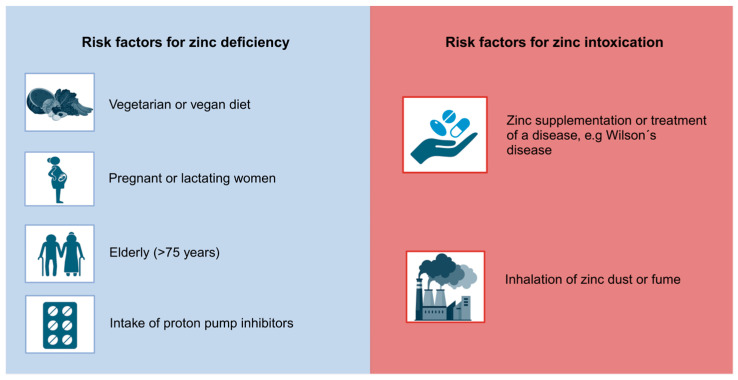
Risk factors for zinc deficiency and zinc intoxication. This figure summarizes Section 3 [26,27,28,29,30,31,32,33,34]. Created with BioRender.com.

Individuals may be at an increased risk of zinc toxicity due to a number of factors. Firstly, those who take excessive zinc supplementation, especially those exceeding the RDA, are susceptible to zinc toxicity [26]. Furthermore, individuals suffering from Wilson’s disease, a rare autosomal recessive disorder characterized by the accumulation of copper, may be predisposed to zinc toxicity, as supplementation of zinc is one treatment option for managing copper levels in the human body. Crucial observation and monitoring of the zinc status are necessary to secure the effectiveness of zinc treatment and to avoid excessive zinc intake [27]. Moreover, individuals employed in industries associated with the process of welding, zinc mining and smelting are at an increased risk of inhaling zinc dust or fume, potentially leading to zinc toxicity [28,29]. An overview of the potential risk factors for zinc intoxication is presented in Figure 1.

### 3.2. Groups at Risk for Zinc Deficiency

Women experience an elevated need for zinc during pregnancy and lactation. This increased requirement arises from the increased nutritional demands of both the mother and the developing fetus, necessitating a higher intake of zinc and other essential micronutrients. According to the WHO, zinc supplementation may help to reduce premature births in a trial involving women of low income [30,31]. Vegetarians and vegans may be at risk for zinc deficiency when compared to an omnivorous diet, as plant-based foods usually contain less bioavailable zinc, as can be seen in Table 1. This can increase the risk of inadequate zinc intake for individuals following this dietary pattern. To counteract the nutritional deficiencies associated with a vegetarian or vegan diet, zinc supplementation can be beneficial [32]. For this reason, an app was recently developed. The Zinc App (https://www.zink-app.de/, accessed on 25 June 2024), developed by Trame et al., allows the determination of the zinc status based on food intake, accounting for both zinc and phytate intake [35]. The app provides the user with an adjusted zinc diet score and indicates whether their zinc status is adequate, deficient or excessive. This free app is particularly useful for individuals following a vegetarian or vegan diet [35,36]. Furthermore, the elderly, especially those above 75 years, are susceptible to zinc deficiency. Besides a typically lower intake of food in this age group, medication such as proton pump inhibitors decrease the absorption of zinc [33,34]. An overview of potential risk factors for zinc deficiency is shown in Figure 1.

## 4. Routes of Zinc

Zinc can be administered through various routes, including dermal (topical) and oral absorption, as well as inhalation. An abnormal high uptake via these three pathways can lead to zinc toxicity.

### 4.1. Dermal

The process of dermal absorption of zinc is a complex topic, as various factors influence the regulation of zinc homeostasis within the dermal layers. Factors that influence the absorption of zinc include the pH level of the skin, the duration and quantity of zinc application and its chemical composition [37,38]. Although dermal absorption of zinc is acknowledged, the precise mechanisms remain unclear.

In a study involving the application of a 25% zinc oxide patch (release rate 5 mg/cm^2^/h) on human skin for 48 h, no evidence of dermal irritation was observed [37,39]. Furthermore, in another research investigation comparing the dermal impacts of different zinc compounds on mice, rabbits and guinea pigs, zinc chloride displayed the highest irritant potency. Zinc acetate induced moderate irritation, while zinc sulfate resulted in low irritant reactions [40]. These findings align with the conclusions drawn by Agren et al., indicating that zinc oxide does not elicit any irritant response on the skin [38]. The irritant response of zinc chloride (solubility in water: 432 g/100 mL) and zinc acetate (solubility in water: 40 g/100 g) can probably be attributed to the higher solubility in water in comparison with zinc oxide (solubility in water: none) [39,41,42]. Topical treatment with zinc oxide increased the mitotic index of epidermal basal cells in mice in a study presented by Jin et al. [43]. Another noteworthy attribute of zinc oxide is its capacity to function as a UV filter in sunscreens, in addition to its ability to act as a photoprotective agent, as exemplified by zinc pyrithione [44,45]. Topical applications of zinc are involved in wound healing due to its anti-inflammatory and antioxidant properties. Moreover, zinc has been found to be beneficial in managing other dermal conditions, like acne vulgaris and skin ulcerations [46,47,48,49]. Research into the topical application of zinc sulfate for viral warts indicates that using a 10% zinc sulfate solution three times a day for a period of four weeks resulted in an 80% reduction in the number of warts [50].

These studies collectively indicate that dermal application of zinc presents minimal to no risk of toxicological reactions.

### 4.2. Oral Intake

While zinc is an essential trace element required for various physiological processes in the body, as mentioned above, excessive amounts of zinc intake can lead to zinc overdose or toxicity. This typically occurs from supplements or denture adhesive creams [51].

The tolerable upper intake level is defined by the EFSA or the National Institutes of Health as the maximum (highest) level of total chronic (daily nutrient) intake that is judged to be unlikely to pose a (likely to pose no) risk of adverse health effects in humans (to almost all individuals in the general population) [52,53]. The UL for an adult (male or female) is set at 25 mg per day, as recommended by the EFSA [14]. However, the UL stated by the FDA is 40 mg per day [13]. Beyond this threshold, there is a risk of impairing copper homeostasis [54,55,56]. It is unlikely that zinc toxicity will occur through dietary intake in a regular diet due to its relatively low bioavailability. However, it is more likely for an individual to have lower serum zinc levels by following a vegetarian or vegan diet compared to those on a non-vegetarian diet [57,58]. In a plant-based diet, higher levels of phytate are consumed, which can hinder zinc absorption. Phytate binds to zinc, forming complexes that cannot be absorbed by the body. Hambridge´s research emphasizes the significance of maintaining a balanced phytate-to-zinc ratio in the diet. By modeling zinc absorption, it was found that the maximal absorption of zinc in adults is approximately 6 mg/day. However, if an individual consumes 1000 mg of phytate per day, the average estimated dietary requirement for zinc doubles [59].

According to several studies, the excessive use of denture adhesive creams, which contain up to 34 mg of zinc per gram of the product, can lead to neurological symptoms and anemia, and can also lead to impaired copper absorption [56,60,61].

If large amounts of zinc-containing creams are used, it is possible that the EFSA UL (25 mg/day) for zinc is exceeded. Similarly, the FDA’s limit can be exceeded if a larger quantity is used. Therefore, the FDA recommends following the instructions on the packaging and to not use more adhesive cream than recommended [62].

Moreover, the concurrent use of oral zinc in combination with quinolone antibiotics (topoisomerase inhibitors) or tetracycline antibiotics (protein synthesis inhibitors) can interfere with the effectiveness in combating bacterial infections. Antibiotics, in turn, may disrupt zinc absorption in the gastrointestinal tract, potentially impairing overall zinc uptake [63,64]. If zinc absorption is compromised, it can weaken the ability of the immune system to function because zinc is crucial for the growth and function of immune cells [65].

### 4.3. Inhalation

Inhalation of zinc is most commonly associated with workplaces in welding, brass plating or hot-dip galvanizing. Workers are exposed to aerosols consisting of particles and gases that include zinc oxide or zinc chloride, leading to potential health risks [66]. Historically, zinc oxide or zinc chloride were used for smoke bombs for military purposes, including in war situations and military trainings [67,68,69].

Inhalation of zinc chloride smoke is a rare but serious trigger for a slowly progressing and frequently fatal condition known as acute respiratory distress syndrome (ARDS) [70]. It was found that soldiers who inhaled hexite smoke (ZnCl_2_) during military training developed ARDS. The soldiers were in critical condition, and attempts were made to prevent the accumulation of collagen in their lungs. Three to four weeks after inhalation, the soldiers died due to severe respiratory failure. Three soldiers in this training group had been wearing masks. They suffered from coughing and dyspnea immediately after exposure. One year later, the lung function tested almost normal [71]. Other studies showed symptoms that come with smoke inhalation, including upper airway obstruction, consolidation and pulmonary edema [72,73,74].

A prolonged exposure to zinc-containing fumes or dust may lead to the development of metal fume fever (MFF). MFF arises from inhaling metal particle-laden fumes, commonly observed among those employed in welding or smelting industries [28,29]. Symptoms typically encompass fever, chills, muscle soreness and respiratory irritation [75,76]. While generally not life-threatening, the discomfort can last a few days [77]. MFF is a reversible disease, but long-term exposure to zinc dust can change the morphology of the lungs, including eosinophilia, goblet cell hyperplasia and pulmonary fibrosis, indicating an inflammatory process [78].

Zinc oxide is defined as a common ultrafine particle in air pollution, as well as a workplace toxin with a size of less than 0.1 µm in diameter. However, it can accumulate into particles with a diameter of 0.1–1.0 µm, which can lead to several health issues when inhaled [79,80].

Monsé et al. discovered that exposure to 1 mg/m^3^ ZnO for 4 h resulted in a dose-dependent acute phase response, characterized by elevated levels of neutrophil granulocytes, serum amyloid A and C-reactive protein in the bloodstream [81].

In mice, it has been demonstrated that the gene expression of interleukin-17f (IL-17f) increases upon zinc oxide inhalation, linking immunological and oxidative stress events within the body. Additionally, elevated gene expression levels of various cytokines, including interferon-γ (IFN-γ), IL-4 and IL-13, have been observed, which may contribute to the progression of long-term allergic asthma [82].

Currently, the Occupational Safety and Health Administration (OSHA) sets the permissible exposure limit for zinc oxide (both dusts and fumes) in workplace air at 5 mg/m^3^ over an 8 h workday and a 40 h work week [83]. In accordance with the guidelines established by the National Institute for Occupational Safety and Health (NIOSH), the maximum allowable short-term exposure limit for a 15 min period is 10 mg/m^3^ [84]. The American Conference of Governmental Industrial Hygienists (ACGIH) sets a threshold limit value (TLV) for zinc oxide at 2 mg/m^3^ for inhalable particulate matter [85]. The German Research Foundation (DFG) sets the maximum workspace concentration (MAK value) for the inhalable fraction of zinc at 2 mg zinc/m^3^, which is also set by the ACGIH. With regard to the respirable fraction, the value is set at 0.1 mg zinc/m^3^ [86].

## 5. Types of Zinc Toxicity: Acute vs. Chronic

There are two main types of zinc toxicity: acute and chronic. Acute zinc toxicity results from an abrupt, high-level exposure to zinc, often caused by consuming large amounts of zinc-containing foods or supplements in a short period of time. Symptoms of acute zinc toxicity include headache, nausea, vomiting, diarrhea, abdominal discomfort and, in rare cases, can also cause metabolic imbalances and severe neurological symptoms [87,88].

Conversely, chronic zinc poisoning occurs gradually over time as a result of extended exposure to elevated zinc levels. This typically occurs as a result of taking supplements containing zinc on a regular basis or experiencing prolonged exposure to zinc dust or fumes at work, as described above. Chronic ingestion of zinc can manifest symptoms associated with zinc-induced copper deficiency, including impaired immune function, decreased levels of high-density lipoprotein (HDL) and increased levels of low-density lipoprotein (LDL) [89]. Furthermore, chronic and excessive use of denture adhesive cream can also lead to zinc-induced copper deficiency and neurological symptoms [61].

## 6. Adverse Symptoms and Side Effects

**Figure 2:**
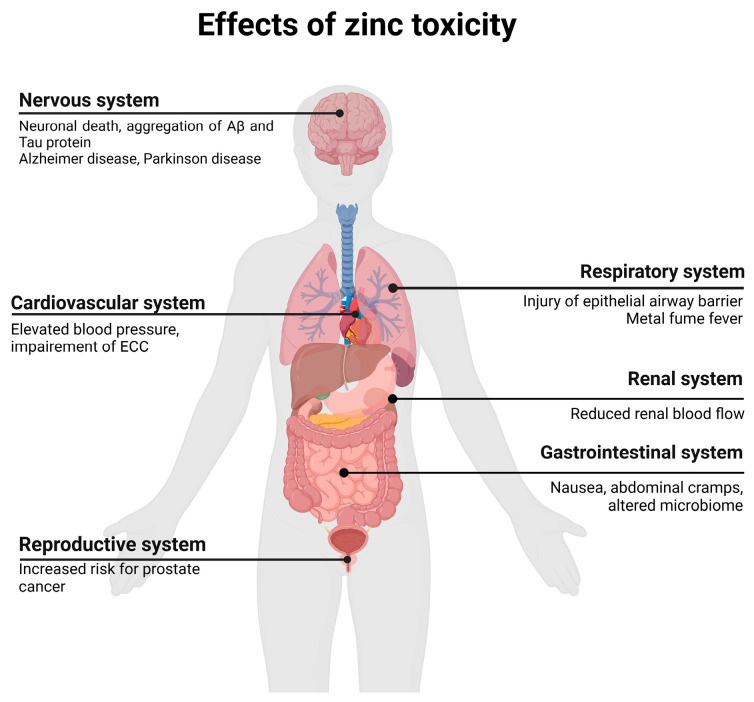
Overview of possible adverse effects and diseases of zinc toxicity. Adapted from “Human organs with Callout”, by BioRender.com (2024). Retrieved from https://app.biorender.com/biorender-templates, accessed on 25 June 2024. ECC, excitation–contraction coupling.

Excessive zinc intake can lead to various adverse symptoms, which are discussed in the following sections according to the respective organ system. An overview of the various side effects and diseases associated with zinc toxicity is shown in Figure 2. Since different zinc salts were used in the mentioned studies, a table was created to display the corresponding content of elemental zinc (Table 6). The differences in elemental zinc content affected the overall concentration required to observe adverse symptoms and side effects. The table also presents examples of studies that reported adverse symptoms and the corresponding concentrations of zinc used.

**Table 6:** Zinc salts with corresponding elemental zinc content and experimental setup.

Zinc Compound	Elemental Zinc	Experimental Setup	Adverse Symptoms	Organism	Reference
Zinc sulfate ZnSO_4_ × 7 H_2_O	22.7%	220 mg zinc sulfate as 50 mg elemental for 6 weeks thrice a day	Nausea, loss of appetite and abdominal cramps	Human	[90]
		0.5 g elemental Zn/L	Serum amylase and lipase levels ↑, 1.5–2 times higher plasma zinc levels, hypertrophied pancreatic islet cells containing more secretory granules	Mice	[91]
Zinc phosphide Zn_2_P_3_	76%	Ingestion of 25 g zinc phosphide (=19 g elemental zinc)	Acute liver failure, acute pancreatitis and death	Human	[92]
Zinc chloride ZnCl_2_	47.97%	>300 µM elemental zinc	Damage to neuronal/cortical structures	In vitro	[93]
Zinc succinate C_4_H_4_O_4_Zn	36.1%	100 mg/kg (=36.1 mg/kg elemental zinc)	Toxic and dystrophic changes in the heart	Mice	[94]
Zinc gluconate C_12_H_22_O_14_Zn	14.3%	65 mg elemental zinc	Undetectable copper levels, neutropenia	Human	[95]
Zinc oxide ZnO	80.3%	Inhalation of 2 mg/m^3^ (=1.606 mg/m^3^ elemental zinc)	Increase of IL-17f, IFN-γ, IL-4 and IL-13	Mice	[82]

### 6.1. Gastrointestinal Effects

Gastrointestinal side effects, including vomiting, stomach ache, diarrhea and nausea, have been reported in animals and humans following acute zinc toxicity [96]. In a study of 47 healthy volunteers, 84% of women and 18% of men reported symptoms such as nausea, loss of appetite and abdominal cramps after the intake of 220 mg zinc sulfate containing 50 mg elemental zinc for six weeks, three times per day. The increased frequency of symptoms in women was attributed to their lower average weight [90]. Patients receiving zinc sulfate therapy, such as those with Wilson’s disease, frequently report adverse gastrointestinal effects, including nausea and abdominal pain, following the ingestion of 45 mg of elemental zinc twice or thrice a day, depending on the body weight and age. In some cases, gastritis with ulcerations or erosion with mild to moderate lymphocyte infiltration were detected [97]. Intoxication with zinc also occurs after ingestion or inhalation of zinc phosphide, which is commonly used as a rodenticide and can cause acute liver failure, acute pancreatitis and death [92]. Even food stored in zinc-galvanized containers can lead to a higher intake of zinc, potentially leading to gastrointestinal side effects [98].

Besides these reported side effects, studies have also revealed molecular changes in the gastrointestinal system after intake of higher zinc levels. In vitro studies have demonstrated that zinc oxide nanoparticles at concentrations of 50 mg/L led to a reduction in gut microbiota and decreased bacterial biodiversity. Excessive zinc doses induce oxidative stress, increase gut permeability, reduce gut wall integrity and induce a shift to pathogenic strains of bacterial pathogens in mice, which could be mediated by elevated free zinc levels available for bacterial pathogens. Nevertheless, normal zinc levels were found to be important for gut bacteria biodiversity and gut wall integrity. The specific effects of high zinc doses are species-specific, as highlighted by Skalny et al. [99]. In experiments, mice were supplemented with zinc for eight weeks. In the mice with a zinc dose of 150 mg/kg bodyweight, decreased production of short-chain fatty acid and fractional inflammatory cell infiltration in the colon were reported. In addition, a high proportion of *Heliobacter hepaticus* was reported, but this was also observed in mice with a low zinc diet (0 mg of supplemented zinc) [100].

Additionally, rare cases of acute pancreatitis have been documented in individuals who have consumed high levels of zinc, with particular attention devoted to investigating the underlying molecular changes [91,92]. After high zinc intake through water (0.5 g elemental Zn/L), the serum amylase and lipase levels were increased in mice. Additionally, the mice had 1.5 to 2 times higher plasma zinc levels and had hypertrophied pancreatic islet cells containing more secretory granules. However, none of these observations had functional consequences [91].

### 6.2. Neurological Effects

Neurological side effects can occur after the oral consumption or inhalation of high levels of zinc. Side effects, including lethargy and hallucinations, have been reported after inhalation [101]. At the cellular level, zinc levels in excess of 300 µM have been shown to induce neurotoxicity [93]. In cortical cell cultures, high zinc levels result in extensive neuronal death, as excessive zinc influx triggers glutathione depletion and ATP loss [102]. As elevated zinc levels are often associated with the death of nerve cells and nerve diseases, these aspects are discussed in more detail in Section 7.2.

### 6.3. Cardio–Renal Effects

A higher intake of zinc has been linked to several adverse effects on both the kidneys and heart, which have also been studied at the molecular level. In vivo, elevated blood pressure and reduced renal blood flow were observed in mice fed a 0.05% or 0.2% zinc-containing diet compared to those observed in mice fed a 0.005% zinc-containing diet. Furthermore, inulin clearance was reduced in a dose-dependent manner, indicating a reduced filtration capacity of the kidneys [103]. In cardiomyocytes, a concentration of 1 µM zinc pyrithione leads to electrical and mechanical dysfunction via reactive oxygen species (ROS) and reactive nitrogen species and dysregulation of calcium, which leads to excitation–contraction coupling impairment. A higher zinc level results in a prolongation of the action potential repolarization phase and a slowing of the process [104]. In mice with a single intragastric zinc succinate dose of 100 mg/kg, toxic and dystrophic changes in the heart were observed one month after treatment [94].

### 6.4. Immunological Effects

Zinc plays an important role in a variety of immunological functions in the innate and adaptive immune systems. Zinc influences immune cell maturation, differentiation and cytokine production. Therefore, zinc deficiency and excess lead to significant changes in the immune system [105]. A previous study showed decreased weight of lymphoid organs and impaired cell cycle in ducks following zinc toxicity. Furthermore, the number of lymphocytes is reduced and the mitochondria in lymphocytes from lymphoid organs are damaged [106]. Excessive zinc levels of 100 µM have been shown to reduce the immune response by suppressing T and B cell functions, as well as IFN-α production and interleukin-1-induced IL-1 receptor kinase (IRAK) activation [107,108]. Mixed lymphocyte culture (MLC) is a common model for allogenic reactions. Zinc concentrations of 60 µM have been shown to suppress alloreactivity in MLC without reducing T-cell proliferation, which further indicates a suppressive effect of zinc on lymphocytes [109].

## 7. Diseases Related to Zinc Toxicity

### 7.1. Zinc-Induced Copper Deficiency

Zinc-induced copper deficiency is a consequence of an excessive dietary intake of zinc, which leads to a reduced absorption of copper in the body, even at low doses above the recommendation of the RDA [110,111].

Copper is an essential trace element. It is involved in processes of energy metabolism, detoxification of reactive oxygen species and iron uptake, as well as cell signaling [112].

A case report by Wazir and Ghobrial highlighted symptoms observed in a patient with copper deficiency, including anemia, leukopenia and myeloneuropathy. The patient’s copper level was found to be undetectably low, falling below the reference range. Oral copper supplementation was administered as treatment, leading to resolution of the anemia and leukopenia within four to six weeks. However, neurological symptoms began to diminish only after six months [113].

Additional case studies have suggested that the symptoms described above were attributed to excessive zinc intake. The termination of zinc supplementation resulted in the resolution of all the symptoms [114,115].

The copper-to-zinc ratio plays an important role in maintaining overall health. The standard value of the serum concentration for copper ranges between 10 and 25 µmol/L, while the serum concentration for zinc is between 12 and 15 µmol/L [116,117]. An imbalance of this ratio may lead to serious diseases and health issues. For example, Wilson’s disease is characterized by copper accumulation. To balance copper levels, zinc supplementation can be used to block the absorption of copper, as mentioned above [27].

Both copper and zinc are absorbed in the small intestine. Zinc homeostasis is regulated by the production of metallothioneins, which are found in enterocytes. Metallothioneins have a high affinity for binding divalent heavy metal ions [118]. Zinc binds to metallothionein at its zinc binding site, acting as an intracellular zinc reservoir. When zinc levels are elevated, the expression of metallothionein is upregulated, serving as a chelating agent [119]. An important characteristic of metallothioneins is their higher affinity for binding copper instead of zinc. Consequently, elevated zinc levels induce the production of metallothioneins, which in turn bind copper, resulting in copper deficiency [55,120]. Excessive zinc intake and copper deficiency were also notable factors in the COVID-19 pandemic. Zinc supplementation is associated with a reduction in the replication of SARS-CoV2, enabling an enhancement of the antiviral immune responses and the support of antioxidant effects [121]. A case study involving a 66-year-old woman with irritable bowel disease revealed undetectable copper levels (<0.10 µg/mL) and elevated zinc levels (2.04 µg/mL) during follow-up, alongside neutropenia. Further investigation unveiled that the patient was ingesting 65 mg of zinc daily by multivitamin and zinc gluconate supplementation, as an alternative to the COVID-19 vaccination. Notably, all zinc intake recommendations and ULs mentioned above were exceeded with the intake of 65 mg zinc per day. To counteract the neutropenia, the patient underwent treatment with filgrastim (granulocyte colony-stimulating factor), the zinc supplementation was discontinued and a copper supplement was started in order to establish adequate zinc and copper levels in the body [95].

The interplay between zinc and copper can also be influenced by other trace elements, such as iron. Both zinc and iron are absorbed in the small intestine using similar transporters, such as divalent metal transporter 1 (DMT1). It was shown that high levels of zinc or iron can inhibit the absorption of the other trace element due to competition for DMT1. Additionally, the efficiency of iron absorption is affected by the presence of copper. Copper is crucial for the function of ceruloplasmin, a protein that facilitates the oxidation of iron to its ferric form (Fe^3+^), thereby enabling its binding to transferrin and transport in the blood. Copper deficiency can impair iron metabolism, resulting in iron accumulation in tissues and anemia. Zinc deficiency can occur if iron levels are excessively high or if the copper absorption interferes with zinc absorption, as mentioned above [9,122].

### 7.2. Neurological Diseases

#### 7.2.1. Zinc in Neuronal Death and Neuronal Diseases

The translocation of synaptic zinc plays a key role in ischemic neuronal death. Zinc-induced apoptosis occurs via a pathway that induces p75^NTR^, thereby triggering caspase activation [123].

Neuronal cell death by zinc showed DNA breaks, indicating cell death by apoptosis, but in electron microscopes, intracellular organelles were swollen and cell membranes were disrupted, indicating necrosis [124]. Vitamin E completely blocks zinc neurotoxicity, leading to the assumption that zinc toxicity affects the production of free radicals [124].

Several diseases are associated with elevated zinc levels and its neurotoxicity, including global ischemia, Alzheimer disease (AD), seizures, Parkinson disease (PD) and multiple sclerosis (MS).

#### 7.2.2. Alzheimer Disease

Alzheimer disease is a chronic neurodegenerative disease and is the most common form of dementia. Alzheimer disease has several molecular pathological hallmarks, including amyloid-β (Aβ) plaque formation and tau phosphorylation, which are major components of neurofibrillary tangles [125]. Blood and cerebrospinal fluid zinc levels in patients with AD have been controversially discussed in various studies [126]. Hu et al. showed that zinc accelerates the abnormal aggregation of human tau and increases tau toxicity in neuronal cells [127]. This stimulation of hyperphosphorylation acts through the inhibition of protein phosphatases (PP2A) and activation of kinases [128]. The aggregation of Aβ into amyloid plaques has been shown to increase 40-fold in the presence of zinc compared to zinc-free Aβ solutions [129].

#### 7.2.3. Global Ischemia

Brain ischemia occurs during stroke or cardiac arrest, and therapy mainly targets the prevention of a high level of irreversible injuries and the restoration of blood flow. The idea that zinc could contribute to neuronal death in this disease was based on the observation that zinc was lost from presynaptic terminals after ischemia and accumulated in degenerating postsynaptic neurons [130]. This elevated zinc accumulation was reported in the first few hours following cerebral ischemia and led to mitochondrial swelling and dysfunction, DNA fragmentation and cell death [131]. Furthermore, zinc can damage the blood–brain barrier (BBB), increasing the permeability of the BBB. These effects, as well as zinc accumulation in microvessels and levels of IL-6, NF-kB p65 and TNF-α, can be reduced by treatment with the zinc chelator Tetrakis-(2-pyridylmethyl)ethylenediamine (TPEN) [132,133].

Ca-Ethylenediaminetetraacetic acid (Ca-EDTA), which acts as a membrane-impermeable zinc chelator, also completely blocked the induction of p75^NTR^ and p75^NTR^-associated death executor (NADE) and the following degeneration of CA1 pyramidal neurons [123].

#### 7.2.4. Parkinson Disease

Parkinson disease is a neurodegenerative condition with symptoms including bradykinesia, rigor and tremor. On a molecular level, PD is defined by the accumulation of α-synuclein in Lewy bodies [134]. Post-mortem studies in patients with idiopathic PD showed increased deposition of zinc in the substantia nigra and striatum, which is characterized by the degeneration of neurons in the substantia nigra and subsequent dopamine loss [135]. In vitro studies in the SH-SY5Y cell line of neuroblastoma markers of dopamine, ROS, DNA damage and mitochondrial dysfunction were used to measure the effects of LC_10_ and LC_50_ concentrations of zinc. The results demonstrated that zinc significantly increases dopaminergic loss, DNA damage and mitochondrial dysfunction [136].

#### 7.2.5. Multiple Sclerosis

Multiple sclerosis is a disease of the nervous system characterized by demyelination, leading to a loss of neurological functions [137,138]. Higher erythrocyte superoxide dismutase (SOD) activity was observed in patients with relapsing–remitting MS, and erythrocyte zinc levels have been shown to be positively correlated with SOD activity, indicating a negative effect of zinc [139]. Furthermore, in multiple sclerosis, zinc can lead to disruption and subsequent immune cell infiltration, damaging white matter in the spinal cord. Administration of zinc chelators decreased these effects. In vivo studies were performed in ZnT3 knockout mice. ZnT3 is responsible for zinc accumulation within synaptic vesicles [140]. This ZnT3 knockout showed protective effects against multiple sclerosis-induced white matter damage and motor deficits, leading to the assumption that extracellular zinc levels are involved in these destructive processes. On the other hand, the overall serum zinc levels in MS patients are found to be decreased in comparison with healthy controls [141,142].

### 7.3. Cancer

The influence of zinc on cancer is controversial. In a study about prostate cancer, 47,240 men were observed over 30 years. Men who used supplemental zinc over 75 mg/day (elemental zinc) were at a higher risk of lethal and aggressive prostate cancer [143]. The prevalence of prostate cancer increases drastically with age, and it has been shown that the zinc concentration in prostatic cells of men over 45 years is ten times higher than in those of men between 18 and 30 years. This elevated zinc concentration in prostatic cells can lead to cellular degeneration and malignant transformation [144]. In addition to prostate cancer, altered zinc levels have been reported in several other cancer types. In some cancers, serum zinc levels are reduced, as in hepatocellular, lung or bladder cancer, but the intracellular concentration can be elevated simultaneously [145]. In other cancer types, such as melanoma, serum zinc levels are increased [146]. It is worth noting that zinc deficiency is a more known aspect to correlate with different types of cancer, including gastric cancer or lung cancer [147,148].

## 8. Prevention and Determination of Zinc Toxicity

An excess of zinc obtained through nutrition is rare, as it is difficult to reach toxic zinc levels through intake of common food, as shown in Table 1 [149]. To prevent excess intake of oral zinc, zinc supplementation should be below the UL [52]. If zinc toxicity is suspected due to oral intake, it can be diagnosed through elevated zinc levels in the blood or reduced copper and ceruloplasmin levels [21]. Additional tools like the Zinc App or radiography can assess zinc status [36,150].

In rare instances, serum zinc levels may reach exceedingly high levels without the manifestation of severe symptoms, a condition known as hypozincemia. For example, a case study described a young girl with a serum zinc level of 128 µmol/L (normal range: 7.65–18.38 µmol/L) despite no zinc supplementation or therapy [151].

In addition to oral intake, inhalation of zinc fumes can lead to zinc toxicity, which primarily occurs in specific industrial settings. To prevent this, it is essential to regulate working conditions in order to avoid inhalation of zinc oxide fumes above the levels recommended by organizations such as WHO, the International Labour Organisation or the German Research Foundation [39,86].

## 9. Conclusions

While zinc is an essential trace element, excessive intake can lead to zinc toxicity, causing adverse effects in the neuronal, gastrointestinal or respiratory system. Furthermore, it can contribute to the development of conditions such as metal fume fever or copper-induced zinc deficiency. Zinc excess is not limited to oral intake but can also occur by inhalation or dermal application of zinc. In the case of dermal application and oral intake, the composition of the zinc salt has a significant influence on the absorption of zinc.

The European Food Safety Authority and the U.S. Food and Drug Administration have set different upper intake levels for zinc at 25 mg and 40 mg per day, respectively. Although the FDA has set the upper intake level to 40 mg per day without expecting toxic effects, a number of studies have indicated that high zinc intake levels can result in adverse effects, including nausea, vomiting, loss of appetite, abdominal cramps, diarrhea and interference with the body’s absorption of essential minerals, such as iron and copper. After reviewing these studies, it appears that the EFSA’s upper intake level of 25 mg per day is more reasonable for avoiding potential negative effects of zinc toxicity.

However, it is important to note that zinc deficiency remains a more common issue than zinc toxicity. Through an analysis of zinc concentration in different foods, it is evident that vegetarians and vegans are particularly susceptible to zinc deficiency, as their diets tend to be lower in zinc-rich foods, such as red meat, poultry and seafood, and higher in phytate level, lowering the bioavailability of zinc. Additionally, as there is no long-term zinc storage in the body, a balance in zinc consumption is crucial to maintain zinc homeostasis and to avoid both deficiency and toxicity.
